# Young People’s Trust in Cocreated Web-Based Resources to Promote Mental Health Literacy: Focus Group Study

**DOI:** 10.2196/38346

**Published:** 2023-01-09

**Authors:** Sachiyo Ito-Jaeger, Elvira Perez Vallejos, Saruka Logathasan, Thomas Curran, Paul Crawford

**Affiliations:** 1 Faculty of Medicine and Health Sciences University of Nottingham Nottingham United Kingdom; 2 National Institute for Health and Care Research (NIHR) Nottingham Biomedical Research Centre Nottingham United Kingdom; 3 Department of Psychological and Behavioural Science London School of Economics London United Kingdom

**Keywords:** trust, mental health, web, young people, cocreation, mental health literacy, qualitative study, thematic analysis, trustworthy, digital mental health, internet, digital health, mobile phone

## Abstract

**Background:**

There is a pressing need to create resources to promote mental health literacy among young people. Digital media is one of the methods that can be used to successfully promote mental health literacy. Although digital mental health resources are generally favorably perceived by young people, one of the essential factors in whether they choose to use these interventions is trust.

**Objective:**

The objective of this study was to explore young people’s trust-related concerns about and recommendations for the cocreated mental health website “What’s Up With Everyone” by using TrustScapes. Our aim was to use the findings to improve the trustworthiness of the website and to inform future creators of web-based mental health resources.

**Methods:**

In total, 30 young people (mean age 19, SD 1.509; range 17-21 years) participated in TrustScapes focus groups. Thematic analysis was carried out to analyze both the TrustScapes worksheets and audio transcripts.

**Results:**

Qualitative analysis revealed that the mental health website contains elements perceived to be both trustworthy and untrustworthy by young people. The relatable and high-quality design, which was achieved by cocreating the website with a team of design professionals and young people, was considered to increase trust. Creators’ credibility also positively affected trust, but the logos and other information about the creators were recommended to be more salient for users. Suggestions were made to update the privacy policy and cookie settings and include communication functions on the platform to improve the trustworthiness of the website.

**Conclusions:**

Factors perceived to be trustworthy included the website's relatable, high-quality design and creators’ credibility, whereas those perceived to be untrustworthy included the privacy policy and cookie settings. The findings highlighted the significance of collaborating with end users and industrial partners and the importance of making the trust-enabling factors salient for users. We hope that these findings will inform future creators of web-based mental health resources to make these resources as trustworthy and effective as possible.

## Introduction

### Background

#### Overview

Mental health problems are increasing globally [1]. Mental ill-health is of particular concern for young people, as 20% of them face mental health issues in any given year [1], and 75% of mental health issues develop by age 24 [2]. The World Health Organization urges increased investment in mental health in all facets, including in mental health awareness, to promote mental health literacy [3]. Mental health literacy is defined as “understanding how to obtain and maintain positive mental health; understanding mental disorders and their treatments; decreasing stigma related to mental disorders; and, enhancing help-seeking efficacy (knowing when and where to seek help and developing competencies designed to improve one’s mental health care and self-management capabilities)” [4]. There is a pressing need to create resources to promote mental health literacy among young people.

#### Digital Mental Health

Digital media is one of the methods that can be used to successfully promote mental health literacy [5-7]. Advancement in digital technology allows mental health information to reach a wide and large audience. Web-based mental health resources have great advantages, such as accessibility, cost-effectiveness, instantaneity, and anonymity [8,9]. They can attract especially young people because such resources are favorably perceived by them and are effective for them [10,11]. According to a recent systematic review of studies from various countries, many young people have had experience in using web-based mental health resources and are prepared to use them [12]. For example, in the United Kingdom, approximately 70% of university students reported that they were confident in using a computer or telephone to seek information about mental health issues [13]. In the United States, 87% of young people aged 14 to 22 years have accessed health information via the web, and 90% of those who had experienced depressive symptoms reported accessing mental health information via the web [14].

#### Trust in Digital Health

Although digital mental health resources are generally favorably perceived by young people, one of the essential factors in whether they choose to use these interventions is trust. Trust is defined as “a positive belief about the perceived reliability of, dependability of, and confidence in a person, object, or process” and is closely related to credibility [15]. It has been described as a relationship between a trustor and a trustee “with optimistic anticipation that the trustee will fulfil the trustor’s expectations” [16]. Trust is imperative in health care, as it is a crucial factor to promote health care access and improve treatment outcomes [17]. Trust is also of great importance when one decides whether to use a particular kind of technology [18] as it is “a precursor to successful and effective adoption, interaction and ongoing commitment in the digital space” [19]. Young people encounter an excessive number of web-based mental health resources, and thus, they face significant challenges in evaluating and selecting which resources to use [20]. As these resources vary largely in their accuracy [20], it is critical that the most informationally accurate web-based resources are perceived as trustworthy by the target users and thus selected for use.

Some factors are found to encourage or discourage users’ trust in digital health. Factors that positively affect trust in digital health resources include stakeholder engagement, creators’ credibility and reputation, validity of the information, fair data access, clear design, and ease of use [9,16,21]. More specifically, engaging stakeholders and taking their suggestions into account appears to be an important condition to increase trust in digital health [16]. In addition, stakeholders are more likely to trust digital health interventions created by public institutions (eg, universities and health services) than those created by private companies (eg, pharmaceutical companies) [16].

In contrast, factors that negatively influence trust in digital health include fear of data exploitation, concerns about privacy, poor information quality, inadequate publicity, excessive cost, and defective technology [9,16,21]. For example, end users find it difficult to trust some digital health systems owing to the fear of data exploitation from third parties [16]. Privacy is one of the major concerns for young people when they access digital mental health interventions [9,22,23].

### Objective

The objective of this study was to explore young people’s trust-related concerns about the cocreated mental health website, What’s Up With Everyone (WUWE; refer to the *Methods* section for details). In addition, we asked young people to generate both realistic and idealistic recommendations to improve the trustworthiness of the website. We aimed to gain deep insight into young people’s perceptions about trust in the web-based mental health resource by using TrustScapes (refer to the *Methods* section for details). Our aim was not only to use the findings to improve the trustworthiness of the website but also to inform the future development of web-based mental health resources.

## Methods

### Ethics Approval

This study was approved by the research ethics committee of the University of Nottingham (application ID CS-2019-R30).

### Participants

After obtaining approval, we recruited an independent sample of young people (ie, different from those who participated in the cocreation process) solely for the purpose of evaluating the website, by distributing flyers and posting the information on a web-based recruitment site. The flyers, containing the description of the study and the eligibility criteria for participation, were distributed via email to mental health organizations, high schools, colleges, and universities in the United Kingdom. As the target audience of the website is a diverse group of young people, we tried to recruit young people from diverse backgrounds to be inclusive and hear diverse opinions. The eligibility criteria for participation included the following: (1) English speaker living in the United Kingdom, (2) aged between 17 and 21 years at the time of recruitment, and (3) has access to the internet and a computer or tablet. Potential participants were asked to submit the Expression of Interest form.

### The WUWE Website

In this study, we explored what elements of the newly created mental health website, WUWE, young people would find trustworthy or untrustworthy, by using TrustScapes (refer to the following sections for details). The WUWE project is a campaign developed to promote mental health literacy among young people. It comprised a series of 5 short, animated films and a companion mental health website created with and for young people, in partnership with multiple award–winning independent animation studio, Aardman Animations.

The website contains information on 5 mental health–related issues commonly experienced by young people (ie, perfectionism, loneliness and isolation, independence, social media, and competitiveness), including the short films corresponding to these themes. The website also contains sections about where to seek help and about the creators, privacy policy, and links to social media. Full details of the project and context can be accessed on the website [24] and the UK Research and Innovation announcement [25]. In a 4-month media campaign following the launch of WUWE on February 8, 2021, the companion website alone attracted 33,100 users, with 44,000 sessions; 101,000 unique page views; 994,000 total page views; and 4500 returning users.

### Stakeholder Engagement

The stakeholders (ie, young people) were actively involved throughout the project, contributing to the development, production, implementation, and evaluation, as recommended by Jirotka et al [26]. Details of how we cocreated the animated films with young people, including how the 5 themes were generated, are described in another paper [27]. Regarding the companion website, 5 mental health professionals with background in psychology or mental health nursing first wrote the text to describe each of the 5 issues. Young people who had been involved in the animation production process then provided feedback on the text (eg, length and complexity) during workshops. The text was revised multiple times until it was fully approved. Young people also provided feedback on the design and layout of the website to the Aardman production team. In the implementation stage, the young cocreators provided suggestions on public relations strategy, such as which influencers are suited for the media campaign. This paper reports the methods and results of the final evaluation stage, focusing on the findings from the TrustScapes focus groups [FGs].

### TrustScapes

The TrustScapes Toolkit was used during FGs to capture young people’s trust-related concerns about and recommendations for the WUWE website. TrustScapes are part of the Fairness Toolkit, which was developed for the UnBias project, funded by the Engineering and Physical Sciences Research Council [28,29]. The Fairness Toolkit is aimed to raise awareness and facilitate a public civic dialogue about how algorithms shape web experiences and to reflect on possible changes to address issues of web fairness [30].

The TrustScapes Toolkit includes a worksheet (Multimedia Appendix 1), keywords (Multimedia Appendix 2), and sketches (Multimedia Appendix 3). The worksheet was cocreated by designers, stakeholders, and researchers through a series of workshops for the stakeholders, including the end users, to visualize their perceptions about data protection, web safety, and algorithmic bias and what they would like to see changed to make the web-based world fair and trustworthy. It is designed to capture both their feelings about the current situation and their dreams and ideals about what the digital world could or should be, in a dynamic and visual manner.

The following four questions are included in the worksheet:

1. Describe an experience of untrustworthiness you are concerned about.
2. Illustrate what is important to you about this experience.
3. How do you think these issues should be addressed by us?
4. Ideally, what would you like to see done?

Keywords and sketches, which had also been cocreated by designers, stakeholders, and researchers, were provided to inspire participants to complete the worksheet and prompt them to contribute their own drawings and insight. TrustScapes are highly interactive and used during FGs to discuss a specific topic (eg, algorithmic systems in mental health).

### Procedure

Young people who confirmed their willingness to participate in the study received and submitted the consent form and a demographic questionnaire via email. A week before the TrustScapes FGs, participants received an email containing the TrustScapes worksheet, keywords, sketches, and the link to the WUWE website and were asked to familiarize themselves with the 3 documents and browse the website before the session. Participants were also asked to have a large piece of paper and a pen for completing the TrustScapes questions.

All TrustScapes FGs were conducted through the web via Zoom, as the United Kingdom was under COVID-19 restrictions at the time of the study. The TrustScapes FGs were moderated by 2 authors who specialize in psychology and digital technologies, and one of them has had substantial experience in facilitating TrustScapes FGs. The TrustScapes FG started with an ice breaker exercise, followed by a description of the TrustScapes methodology and the aims of the study.

A moderator showed the WUWE website to the participants by sharing their computer screen. Participants were asked to discuss trust-related concerns about and recommendations for the website on each page (ie, cookie pop-up, home page, about us, privacy policy, theme pages such as perfectionism, seeking help, and social media such as Instagram). Following the group discussions, participants were asked to complete the TrustScapes worksheet independently. Once all participants completed the TrustScapes worksheet, each participant presented their ideas to the whole group. Participants emailed their TrustScapes worksheet to the moderator after the session. Each session lasted for 1.5 hours and was audio-recorded and transcribed verbatim. Participants were compensated financially for their participation. A total of 6 TrustScapes FGs were conducted, and each session consisted of 3 to 7 participants. Data saturation became apparent, with major trends being clear by the end of the sixth FG; thus, further sessions were not conducted.

### Data Analysis

Thematic analysis was used to analyze the data collected from the TrustScapes worksheets and transcripts [31]. Thematic analysis has been used to understand young people’s opinions about a newly developed digital intervention [27,32]. Following the six phases of thematic analysis by Braun and Clarke [31,33], an author (1) became familiar with the content by reading and rereading the transcripts, (2) generated initial codes, (3) searched for themes, (4) reviewed the themes, (5) defined and named the themes, and later, (6) produced the report. Throughout the process, research meetings were conducted for debriefing and discussion among the authors.

## Results

### Participant Characteristics

In total, 30 young people (mean age 19, SD 1.509; range 17-21 years) participated in the study. More than 70% of the participants were women. Participants were from diverse ethnic backgrounds, with White British being the majority (7/30, 23%). Approximately half of the participants (14/30, 47%) had level-3 qualification as their highest level of education, and 30% (9/30) had level-2 qualification. Complete demographic information is presented in Table 1.

**Table 1:** Demographics of participants (N=30).

Characteristics				Participants, n (%)
**Sex**				
	Female			22 (73)
	Male			7 (23)
	Prefer not to say			1 (3)
**Ethnic background**				
	**White**			
		English, Welsh, Scottish, Northern Irish, or British	7 (23)	
		Irish	1 (3)	
		Other	3 (10)	
	**Multiple ethnic groups**			
		White and Black Caribbean	1 (3)	
		White and Black African	1 (3)	
		Other	1 (3)	
	**Asian**			
		Indian	5 (17)	
		Pakistani	1 (3)	
		Bangladeshi	2 (7)	
		Other	2 (7)	
	**Black, African, Caribbean, or Black British**			
		African	2 (7)	
	Other			1 (3)
	Prefer not to say			3 (10)
**Religion**				
	Christian			6 (20)
	Hindu			2 (7)
	Muslim			4 (13)
	Sikh			1 (3)
	Prefer not to say			6 (20)
	Other			1 (3)
	None			10 (33)
**Highest level of qualification^a^ (participant)**				
	None			0 (0)
	Level 1^b^			2 (7)
	Level 2^c^			9 (30)
	Level 3^d^			14 (47)
	Level 4 or above^e^			2 (7)
	Other^f^			1 (3)
	Prefer not to say			2 (7)
**Highest level of qualification^a^ (parent, guardian, or carer)**				
	None			2 (7)
	Level 1^b^			1 (3)
	Level 2^c^			4 (13)
	Level 3^d^			3 (10)
	Level 4 or above^e^			8 (27)
	Other^f^			4 (13)
	Prefer not to say			8 (27)

^a^Qualification levels (UK census).

^b^Includes O levels 1-4, Certificate of Secondary Education (CSEs), or General CSE (any grade); entry level; Foundation Diploma; National Vocational Qualification (NVQ) level 1; Foundation General NVQ (GNVQ); and basic skills.

^c^Includes O level ≥5 (pass), CSEs (grade 1), or General CSEs (grades A*-C); school certificate; A level 1, Advanced Supplementary level 2-3, or Victoria Certificate of Education; higher diploma; NVQ level 2; Intermediate GNVQ; City and Guilds Craft; Business and Technology Education Council (BTEC) First or General Diploma; and Royal Society of Art (RSA) Diploma.

^d^Includes A level ≥2 or Victoria Certificate of Education, Advanced Supplementary level ≥4, higher school certificate, Progression or Advanced Diploma, NVQ level 3, Advanced GNVQ, City and Guilds Advanced Craft, Ordinary National Certificate, Ordinary National Diploma, BTEC National, and RSA Advanced Diploma.

^e^Includes degree (eg, BA and BSc), higher degree (eg, MA, PhD, and Postgraduate Certificate in Education), NVQ level 4-5, Higher National Certificate, Higher National Diploma, RSA Higher Diploma, BTEC higher level, and professional qualifications (eg, teaching, nursing, and accountancy).

^f^Includes other vocational or work-related qualifications and foreign qualifications.

### Themes and Subthemes

#### Overview

**Figure 1:**
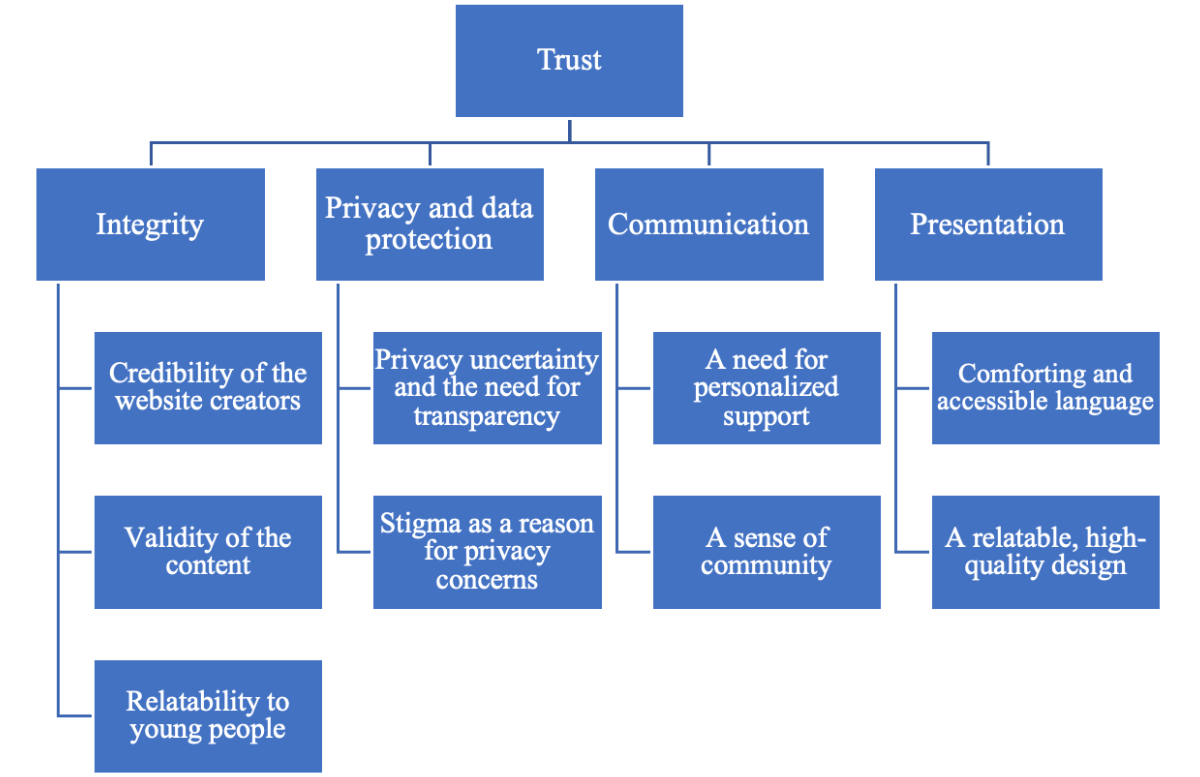
Themes related to young people’s trust in the cocreated mental health website.

After applying thematic analysis across the 2 data sets (TrustScapes worksheets and FG transcripts), 4 main themes and 2 to 3 subthemes for each theme were generated (Figure 1). The 4 main themes were integrity, privacy and data protection, communication, and presentation. Each subtheme is described below, with supporting quotes from participants’ TrustScapes worksheets and FGs. The quotes from the 2 data sets were complementary, as the same themes and subthemes were generated from the 2 separate analyses.

#### Integrity

The theme of integrity highlights that the credibility of the website creators, validity of the website content, and relatability to young people are important aspects to enhance trust for the end users.

##### Credibility of the Website Creators

A major factor that created a sense of trust among participants was the involvement of established institutions and organizations in the development of the WUWE website. Many participants felt that the inclusion of logos helped reassure them that the website had good intentions and the information on it could be trusted:

Trusting the website is the most important part as someone experiencing issues may not believe that the advice this website is offering is true or valid and having that trust, which can be gained from the organisations involved.

Participant 8; FG2; worksheet

However, some suggested the link to the *about us* page, which contains the logos, needs to be more salient (eg, by moving the button to the top of the landing page and making the button large) for easy access of information. Another suggestion was made to move all the information on the *about us* page to the landing page so that users could see that the WUWE website was legitimate as soon as they access the website:

[About Us] should be incorporated into the landing page so users know what to expect or gain from the website. Otherwise, there might be a lot of anxiety around the purpose of the website.

Participant 10; FG2; worksheet

##### Validity of the Content

Similarly, some participants recommended to include the source of the information on the website so that the end users can trust the content. In particular, it was suggested to indicate whether the information on the website had been proven to be significant or effective:

As a student I’m more trusting of ideas/findings that have been established as effective/significant in research studies. On the pages for each theme, if an idea/suggestion for how to deal with an issue is presented it’d be nice for a word in the sentence to be highlighted and have a hyperlink to the original study that this idea came from.

Participant 22; FG5; worksheet

##### Relatability to Young People

Some participants indicated that the information that young people were involved and considered in the creation of the website helps them to trust the content of the website. As this information was written only briefly on the *about us* page, participants recommended to highlight the information:

I think [knowing that WUWE was cocreated with young people] increases trust because it shows that you really care about the website because it is not just like adults only trying to make something for young people, you have actually interacted with young people to produce something that they want so I think that...it brings like reliability almost.

Participant 28; FG6; transcript

#### Privacy and Data Protection

The following 2 subthemes highlight that privacy is one of the major concerns for young people when they access web-based mental health resources.

##### Privacy Uncertainty and the Need for Transparency

Many participants were concerned about privacy. As they would not read the privacy policy owing to its length and complexity, they felt that they may be consenting to something they do not understand. They were particularly worried about their data being sold to third parties without their knowledge:

The privacy policy is quite long, you may consent to something you didn’t read.

Participant 17; FG4; worksheet

Understanding the information easily [is important to me] - not feeling like I’m drowning. Illustration of a person drowning.

Participant 18; FG4; worksheet

I am worried the website would access features such as my location. The website may decide to sell my data in the future. Original illustration of a person and a computer.

Participant 16; FG 4; worksheet

Participants highlighted that these issues of distrust could be solved by increasing the transparency between the website and end users. They wanted to be reassured that data were not being sold to third parties and wanted to know whether their data were being stored:

Make clear what information will be taken whether it will be used by any third parties. TrustScapes illustrations of exploiting data and leaking data.

Participant 9; FG2; worksheet

TrustScapes illustrations of data mining, digital surveillance, and being watched.

Participant 27; FG6; worksheet

Similarly, many participants felt that the website needed more clarity about which cookies are being used and how exactly a user’s data are managed and used. They recommended that users should have more control over the cookies that are being used. The WUWE website has only 2 cookie options: *allow cookies* and *decline*. Young people proposed that users should be provided with the option to select only the essential cookies or those they wanted to activate, so that the users have more control over their data:

Make it clear and transparent if cookies are/are not being used, what type of cookies, option to accept/reject specific cookies.

Participant 1; FG1; worksheet

Give a selection of cookies to accept rather than Accept All.

Participant 16; FG4; worksheet

At the time of the TrustScapes FGs, one of the cookie options, *allow cookies*, was more visually salient (ie, in a black box) than *decline* (ie, in a white box), and thus, the users’ attention was more likely to be drawn to *allow cookies*. Participants urged for the design to be changed to present the 2 options in a similar manner. The design has since been modified after reviewing the participants’ recommendation:

The Cookie pop-up at the start is quite leading towards accept rather than decline. Make both accept and decline bold boxes.

Participant 8; FG2; worksheet

##### Stigma as a Reason for Privacy Concerns

Young people voiced the importance of privacy and need for anonymity, in part owing to the stigma around mental health issues. Some participants recommended to include a function that would allow users to quickly exit or hide the website:

Some people are quite defensive about their mental health issues, and so may not want those around them to know about this.

Participant 7; FG2; worksheet

Having a way to quickly hide the page, so that the user can cover what they’re looking at, but can quickly go back to it, if they wish to –may want to hide the page from family walking past, for example, if they’re not wanting to share their feelings with their family at that moment in time.

Participant 4; FG2; worksheet

It could be a concern for a young person struggling to talk about their mental health that if they accept cookies on say a family computer, WUWE may follow them to other sites and expose their search history that they’re not comfortable sharing.

Participant 27; FG6; worksheet

#### Communication

The following 2 subthemes involve participants highlighting the need to be able to communicate with others to not feel alone on the internet.

#### A Need for Personalized Support

A concern raised about the WUWE website was the lack of personalized help and responses. Some participants felt that it would be easy to trust a website if live chat features were included for instant support:

Install and set up an online chat bot system—would provide instant help.

Participant 3; FG1; worksheet

This would allow users to have someone to talk to, and they will not feel alone. Participants highlighted that having professional support, such as therapists, would be a helpful inclusion and ultimately help the user to trust the remote resource:

An online 24/7 Live Chat facility in order to feel that there are people behind the organisation to make it more trustworthy, and that the person using the website is being considered at every touchpoint.

Participant 21; FG5; worksheet

#### A Sense of Community

Similarly, some participants reported that it would be helpful to have a function on the platform to interact anonymously with other young people about their mental health. Ideas for how this could be achieved included adding a space to leave comments. It was also highlighted that the inclusion of social media makes the WUWE campaign more accessible to young people, thus helping to create a positive environment:

Have a place to let others put comments and issues they have and allow everyone to interact with each other.

Participant 24; FG5; worksheet

I think [social media] just makes it a lot more accessible to people and I think that erm it is definitely something positive that people can see.

Participant 1; FG1; transcript

Suggestions were made to include quotes from young people who have benefited from the website. Trust is increased if the quotes include the person’s identity, such as their name, photo, or avatar:

I think that giving names or posting photos from those helped by this service may be beneficial.

Participant 25; FG5; worksheet

#### Presentation

The following 2 subthemes highlight how the presentation of a website influences whether a young person decides to trust the website.

##### Use of Comforting and Accessible Language

The language used on the WUWE website played an important role in whether users trusted the web-based mental health resource. Some participants felt that the repeated use of the word *help* was overwhelming and that the website needed more comforting language to create a safe web-based environment:

Change “help” to “support” or have a heading like “Where can I go”; Change “About us” to “Come and meet us” or “Who we are.”

Participant 3; FG1; worksheet

Related to the subtheme, privacy uncertainty and the need for transparency, many participants mentioned that the language used for the privacy policy was very difficult to understand. It was suggested that WUWE should consider providing a version with more accessible language or presenting it using an animated video to help more users understand and feel comfortable with what they are agreeing to:

I think it is important the privacy policy is less automated and includes accessible language that everyone is able to understand so they know exactly how their data is being stored. Include an animated video about the privacy policy, create a separate privacy policy with easy-to-understand language.

Participant 19; FG4; worksheet

Shorten the privacy policy and have 2 versions; short and long.

Participant 16; FG4; worksheet

##### A Relatable, High-Quality Design

Many participants felt that the design of the website was relatable to young people and had high production quality. In particular, the color scheme and use of animations made the experience more comforting. They felt this was a good way to present information in a light-hearted and approachable manner:

I found the colour scheme particularly trustworthy because I feel like this made the experience more relatable to a young audience, also the use of the animations and doodles around the pages made the pages feel comfortable and easy to read.

Participant 28; FG6; worksheet

I think this is more like almost a light-hearted way of laying everything out.

Participant 2; FG1; transcript

Related to the subtheme, relatability to young people, many participants reported that the cocreation of the website with young people helped the design to be relatable to young people:

I think the layout kind of...and the colour scheme erm and you know like the fact that there are videos voiced by you know young people and things like that, I think that kind of tells you [young people were involved].

Participant 3; FG1; transcript

Many participants felt that the high-quality design of the website was a strong trust enabler, as it shows users that a lot of thought has been put into the creation of this website. This makes the website appear more professional and, therefore, more trustworthy:

I think it makes it a bit more trustworthy because it shows that more thought has gone into it...the fact that it is so professionally done, it makes it seem more legitimate.

Participant 27; FG6; transcript

## Discussion

### Principal Findings

This study explored young people’s trust-related concerns about the mental health website. Qualitative analysis revealed that the WUWE website contains elements perceived to be both trustworthy (eg, relatable, high-quality design and creators’ credibility) and untrustworthy (eg, privacy policy and cookie setting) by young people. Although some of the factors influencing trust in this study were complementary to those in previous studies, TrustScapes allowed us to gain deep insight into young people’s perceptions about trust in mental health resources, including how to improve the trustworthiness of the website.

The fact that the website was created through collaborations among the established institutions and organizations, young people, and a professional creative studio contributed to increased trust in the website. Young people perceived that the high production quality and colorful and comforting design of the website increased trust. The relatable, high-quality design was achieved by collaborating with the end users and a team of design professionals. Stakeholder engagement is one of the pillars of responsible research and innovation and is crucial for creating new media interventions relatable to end users [26]. This study highlights the importance of stakeholder engagement and interdisciplinary collaborations with industrial partners.

Consistent with previous studies [9,16,21], the creators’ credibility positively affected trust in digital health. In this study, although the website includes the information about the established institutions’ involvement in the study, the information was recommended to be more salient. According to the Prominence-Interpretation Theory, it is not possible for users to notice all elements of a website, and as a result, not all elements are evaluated for credibility [34]. Therefore, website creators should ensure that the elements found to increase trust, such as logos of established institutions, are salient for users.

Consistent with previous studies investigating trust in digital health, privacy is of particular concern for young people [16,22] and transparent privacy policies are recommended to increase trust in a cocreated web-based platform [35]. Some suggestions were made to reduce privacy-related concerns and thus increase trust in the mental health website. One suggestion was to make the privacy policy easy to comprehend for young people. Inspired by this project, a proposal was made to use animations to explain complex privacy policies in clear language. Previous studies support the idea that animation is effective in communicating complex information in an understandable manner [36-39]. Thus, future web service providers should consider using animations to explain privacy policies.

Another way to increase trust related to privacy is to provide cookie settings that give more control to users. Users should be given the option to select the cookies that they wish to activate rather than only limited options (eg, *allow cookies* and *decline*). Moreover, cookie settings should not be designed to lead users to one of the options (ie, *allow cookies*) over the other (eg, *decline*). This is consistent with the age-appropriate design code, which requires web service providers to not use *nudge techniques* that encourage children to turn off privacy protections [40]. Although the code only covers web-based services that are likely to be accessed by children, this practice should be extended to those targeted at young people.

Stigma related to mental ill-health appears to be one of the reasons why privacy is an important factor associated with trust in digital mental health. Stigma is one of the most significant barriers to in-person mental health help seeking among young people [41]. Digital health interventions have the advantage of anonymity if their privacy is well protected [9]. In addition to the methods listed previously to protect users’ privacy, young people proposed to have a function to hide the mental health website page from their family or friends, as some users may not be ready to reveal to others that they have mental health concerns. Although we should aim to reduce the stigma itself by promoting interventions such as the WUWE animations and website, functions to reassure young people of their privacy and anonymity should be incorporated.

Recommendations were made to include functions on the platform that enable users to communicate with other young people or mental health professionals, as shown in previous studies [16]. The WUWE website includes a *help seeking* page, which contains the details of a wide variety of mental health organizations with which young people can communicate via text or email, on the phone, or in person. However, this study revealed that young people prefer such communication tools integrated into the website, which allows them to feel more connected to the website and thus increase trust. Future creators of digital health interventions may include communication functions on the platform by collaborating with mental health organizations that are already providing such services.

Finally, it is important to young people that web-based mental health resources are *comforting*. Regarding the design, consistent with the previous studies on youth digital interventions [42,43], young people reported liking the pleasant and comforting design. Similarly, our participants suggested that the language used on the website needed to be more comforting. For example, the use of the word *help* is perceived to be overwhelming and thus needs to be reconsidered. The word *help* is frequently used in mental health. *Help seeking* is one of the components of mental health literacy [44]. For young people to feel more comfortable and safe, especially when they are distressed, mental health professionals may consider using the word *support seeking* instead.

### Limitations and Future Studies

Several limitations of the study should be noted. First, the sample consisted mostly of female participants. Although we aimed to recruit more male participants by exclusively recruiting them through a recruitment website, we gained only limited interest from men. Second, because of the COVID-19 restrictions during the study, we conducted all TrustScapes FGs via the web. Although 96% of all households in the United Kingdom have internet access and 99% of young people in the United Kingdom have a smartphone [45,46], it is possible that some young people with limited access to technology were prevented from participating in the study. To be more inclusive, future researchers should conduct FGs both in person and via the web.

Third, owing to time constraint, the TrustScapes FGs were conducted only after the website was promoted to the public. Although our young cocreators (ie, young people who cocreated the animations and website) provided valuable feedback and some adjustments were made accordingly before the launch, it is apparent that the recommendations from the independent sample of young people (ie, the TrustScapes participants) would have made the website even more trustworthy. We were able to make a few changes to the WUWE website based on the findings from TrustScapes (eg, cookie design) so far, but we recommend that future projects should allocate sufficient time before the public launch to adjust the intervention based on feedback from an independent group of end users (ie, those who were not involved in the cocreation process).

### Conclusions

This study provided insight into young people’s trust-related concerns about and recommendations for cocreated web-based mental health resources. Factors perceived to be trustworthy included the relatable, high-quality design and creators’ credibility, whereas those perceived to be untrustworthy included the privacy policy and cookie settings. The findings highlighted the significance of collaborating with end users and industrial partners and the importance of making the trust-enabling factors salient for users. We hope that these findings will inform future creators of web-based mental health resources to make them as trustworthy and effective as possible.

## Appendix

Multimedia Appendix 1 TrustScapes worksheet.

Multimedia Appendix 2 TrustScapes keywords.

Multimedia Appendix 3 TrustScapes sketches.

## Abbreviations

FG: focus group
WUWE: What’s Up With Everyone

## Data Availability

Anonymized focus group transcripts are available from the University of Nottingham data repository [47].
