# Supplementary material for: Young People’s Trust in Cocreated Web-Based Resources to Promote Mental Health Literacy: Focus Group Study
Source: JMIR Ment Health. 2023 Jan 9;10:e38346. doi: 10.2196/38346 (PMC9871878; doi:10.2196/38346)
Supplement: Multimedia Appendix 2 [file mental_v10i1e38346_app2.pdf]

access	accountable	acknowledged	addiction
allowed	ambiguous	anxiety	attached
attention	automated	aware	bad
bandwagon	barriers	behaviour	being followed
belief	bias	buying	cage
central	certain	certify	change
choice	claim	clear	closed
confined	comfortable	common	compromise
conformity	confusion	connection	control
convenience	danger	deception	definitive
denial	depth	detached	different
disbelief	disruption	distraction	dynamic
echo chamber	empowered	enabled	encryption
enemy	escape	exploited	fact
fair	fake news	false	familiar
fiction	filter	filter bubble	following
formal	free	freedom	friend
genuine	good	gossip	hacked
handcuffs	health	hidden	hollow
ignorance	ignored	illegal	illness
informal	intervention	justice	knowledge

UNBIAS

This work is licensed under a Creative Commons: Attribution Non-Commercial Share-Alike 4.0 International Licence.

Concept & Design proboscis.org.uk

proboscis

lawful	laws	legal	license
lies	location	locked up	loopholes
loss	magnetic	manipulation	marginal
meaningless	mistrust	moderate	monitored
necessary	negative	normal	obscure
open	owned	permission	personal
positive	power	pressure	prevented
privacy	private	profit	protection
protest	puppet	ratings	rebellion
reality	recommend	regulations	released
reliable	representation	reputation	restriction
review	risk	rubbish	rules
rumour	safety	secret	selling
shared	social	solid	standard
static	status quo	stereotyped	strange
stress	substantial	suggestion	superficial
surface	surveillance	suspicion	swamped
tracked	transparency	true	trust
truth	unaware	uncertain	uncomfortable
uncommon	unfair	unfamiliar	unlawful
unnecessary	verifiable	virtual	well-being

UNBIAS

This work is licensed under a Creative Commons: Attribution Non-Commercial Share-Alike 4.0 International Licence.

Concept & Design proboscis.org.uk

proboscis
