# Supplementary material for: Young People’s Trust in Cocreated Web-Based Resources to Promote Mental Health Literacy: Focus Group Study
Source: JMIR Ment Health. 2023 Jan 9;10:e38346. doi: 10.2196/38346 (PMC9871878; doi:10.2196/38346)
Supplement: Multimedia Appendix 3 [file mental_v10i1e38346_app3.pdf]

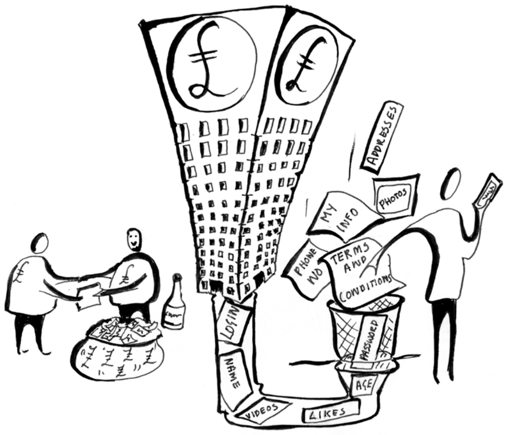

EXPLOITING DATA

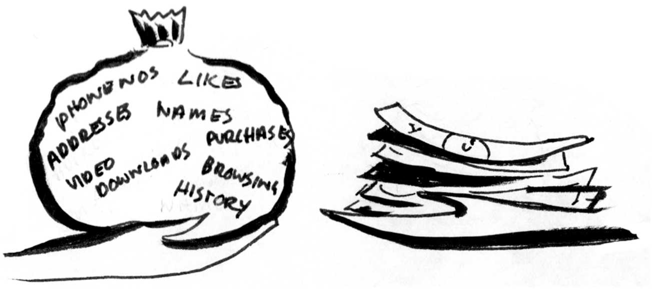

SELLING DATA

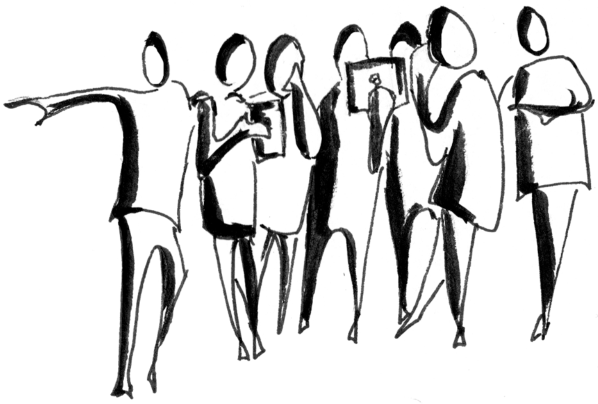

GOSSIP / RUMOUR

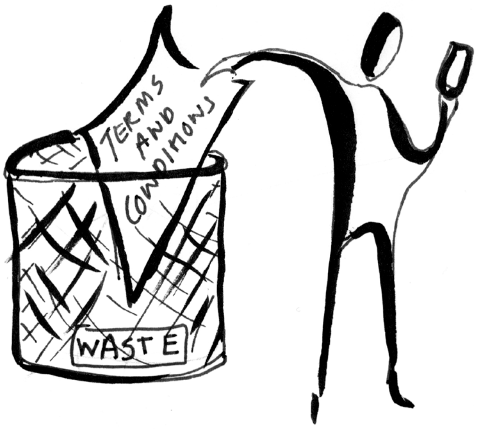

IGNORING T&Cs

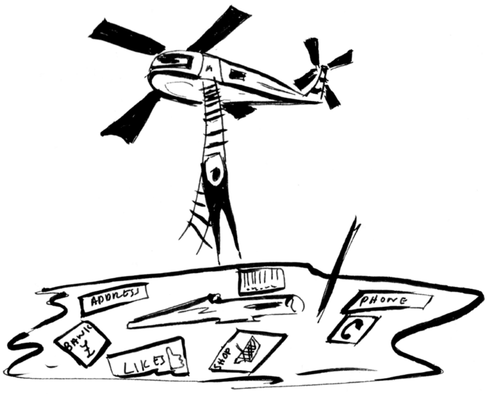

RESCUE

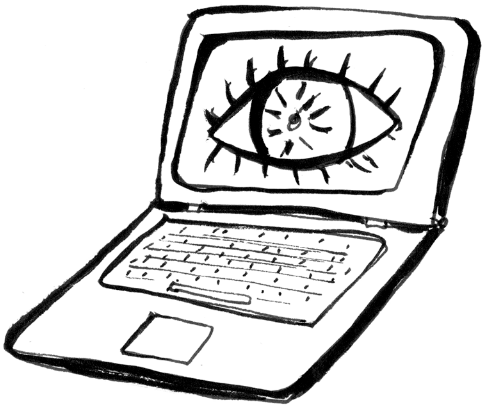

DIGITAL SURVEILLANCE

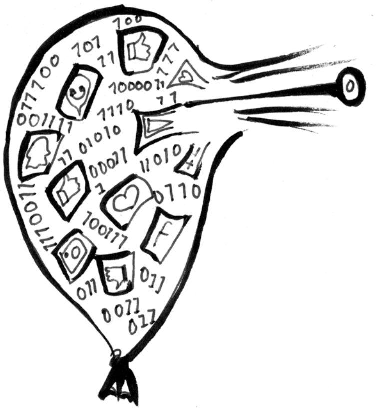

BURST THE BUBBLE

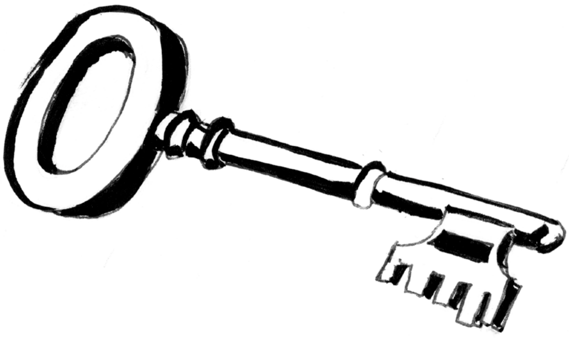

KEY

UNBIAS | This work is licensed under a Creative Commons: Attribution Non-Commercial Share-Alike 4.0 International Licence. | Concept & Design proboscis.org.uk | proboscis

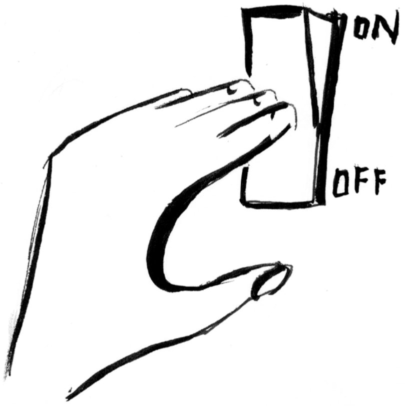

SWITCHING OFF

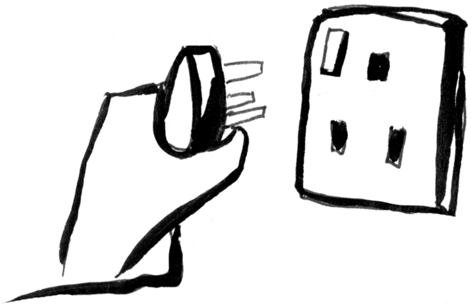

PULLING THE PLUG

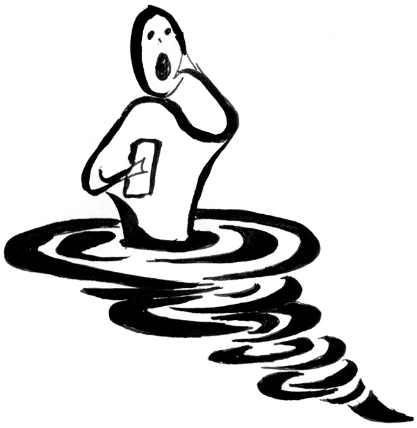

WHIRLPOOL

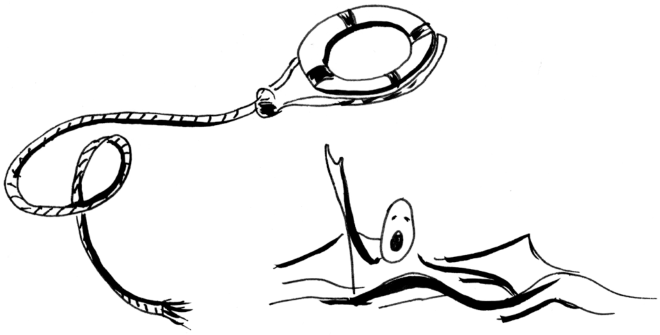

LIFELINE

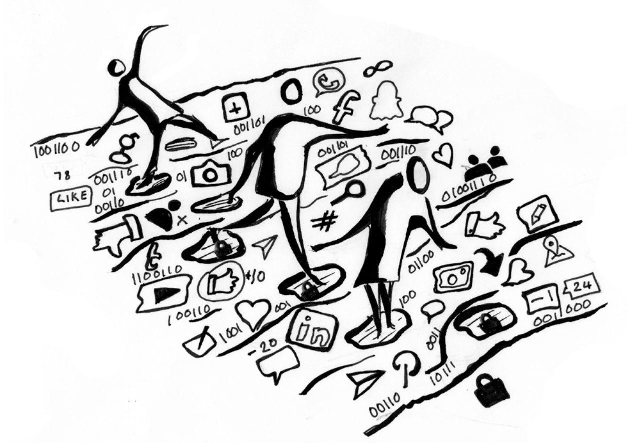

STEPPING STONES

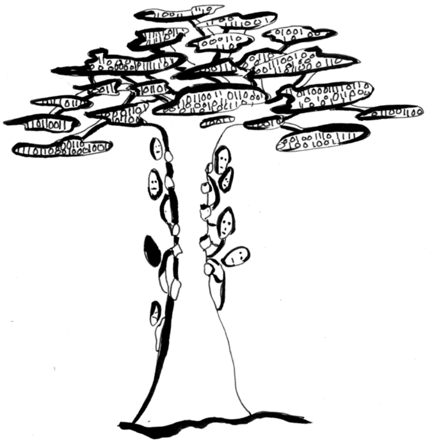

BEING WATCHED

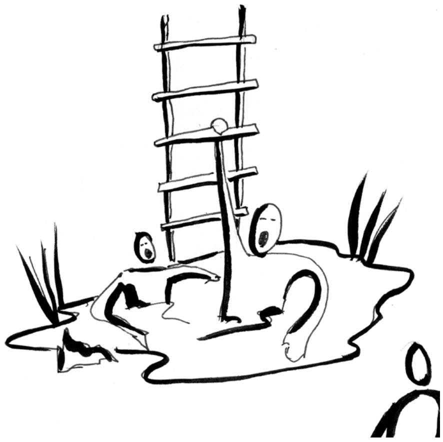

DIGITAL SWAMP

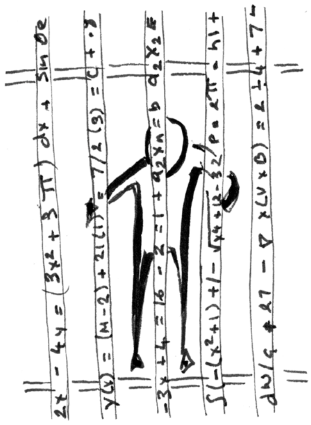

CAGE / PRISON

This work is licensed under a Creative Commons: Attribution Non-Commercial Share-Alike 4.0 International Licence.

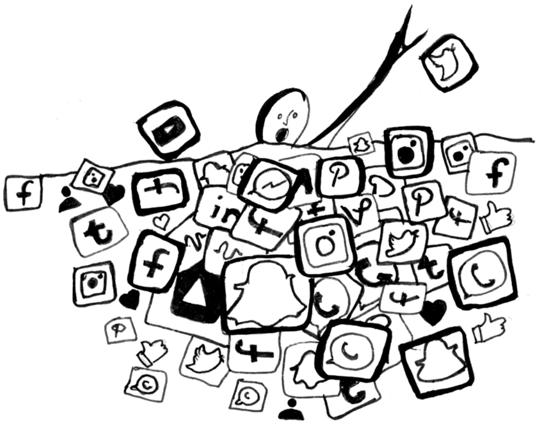

DROWNING IN APPS

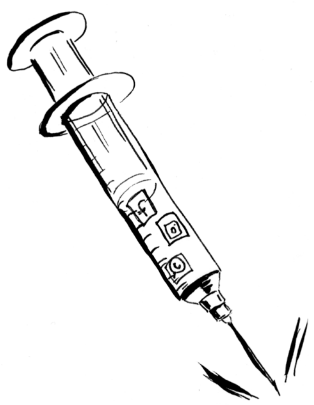

ADDICTION

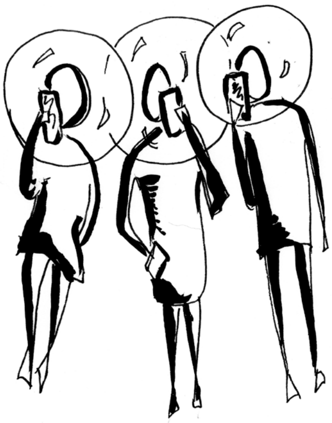

FILTER BUBBLE

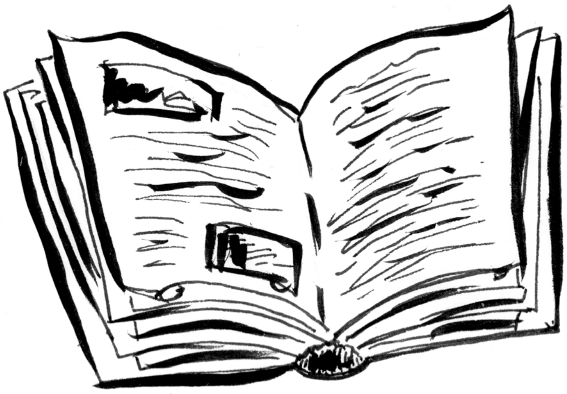

OPEN BOOK

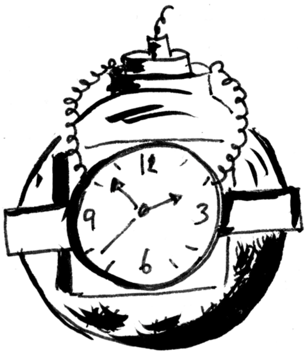

TIME BOMB

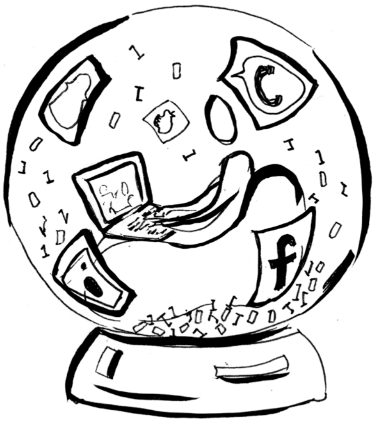

ECHO CHAMBER

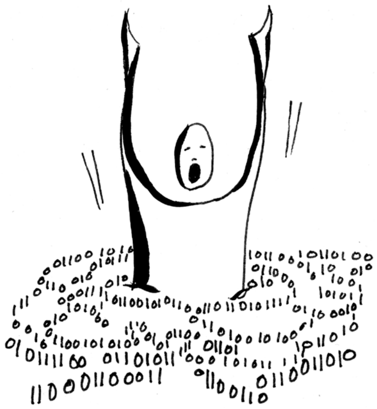

DATA OVERLOAD

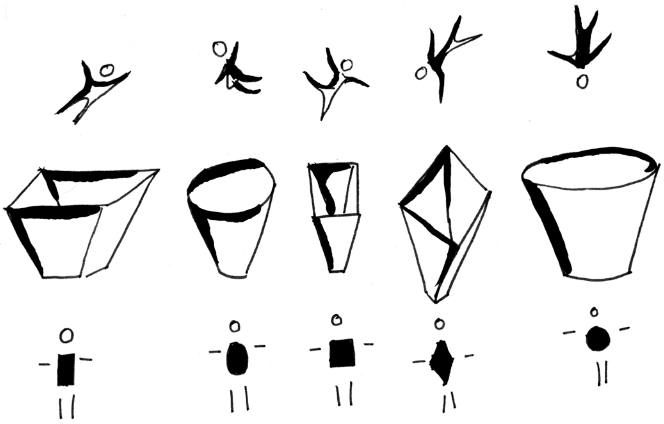

BEING PROFILED

UnBIAS
This work is licensed under a Creative Commons: Attribution Non-Commercial Share-Alike 4.0 International Licence.
Concept & Design proboscis.org.uk
proboscis

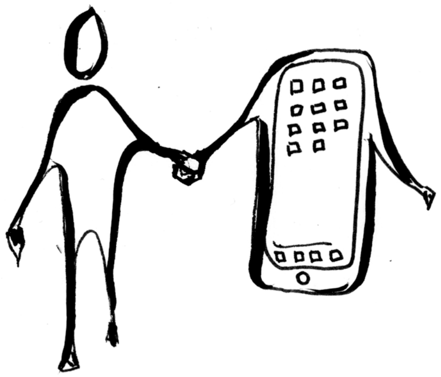

DEVICE AS FRIEND

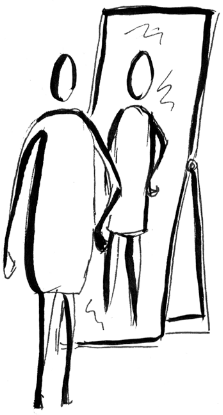

MIRROR

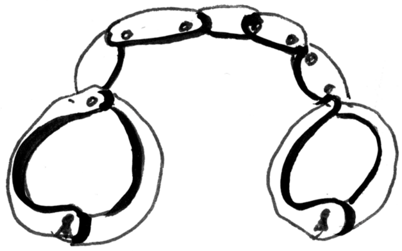

HANDCUFFED / CHAINED

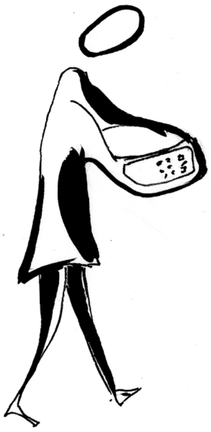

DIGITAL LIMB

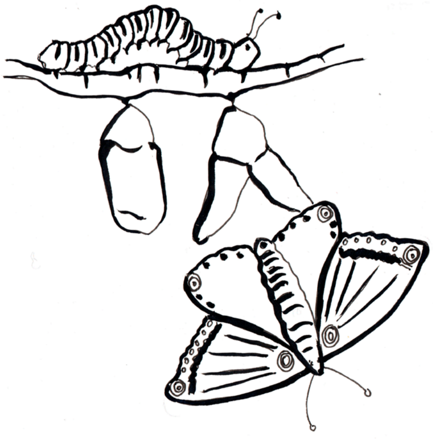

TRANSFORMATION

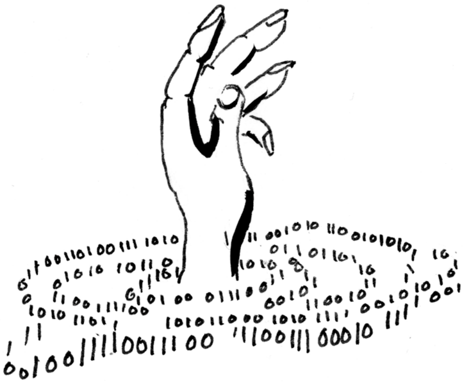

SINKING

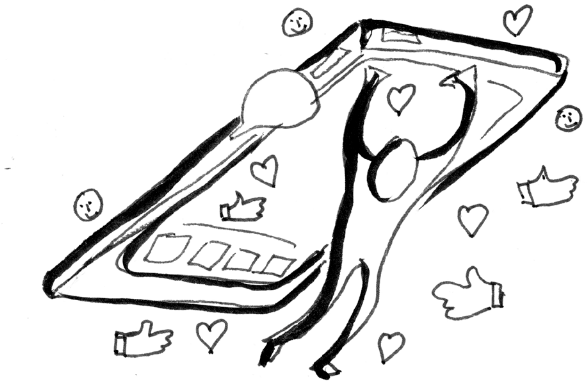

CRUSHED

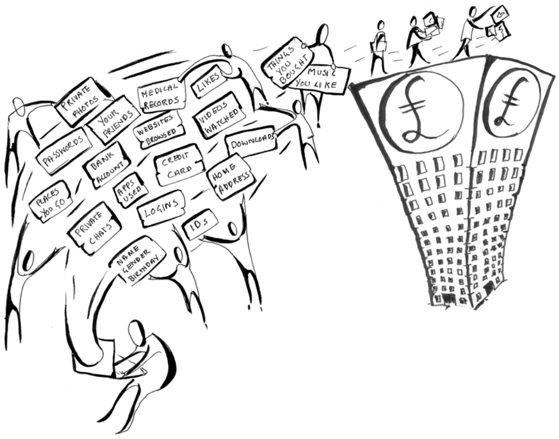

DATA MINING

This work is licensed under a Creative Commons: Attribution Non-Commercial Share-Alike 4.0 International Licence.

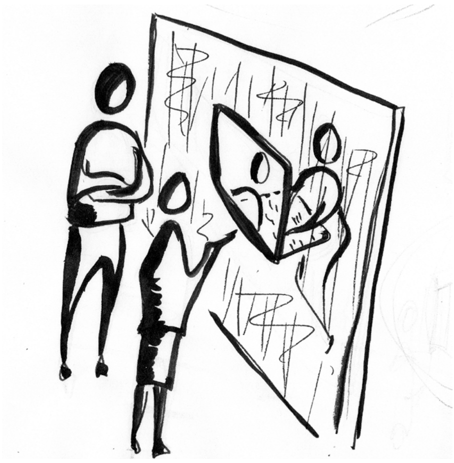

TWO-WAY MIRROR

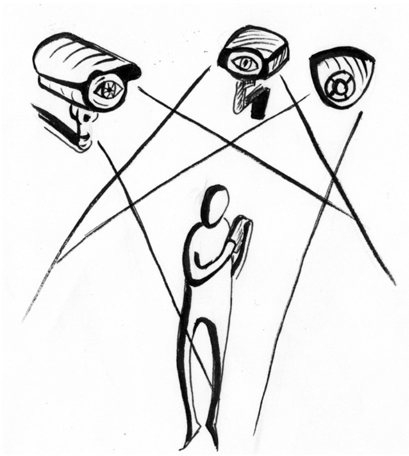

BEING WATCHED

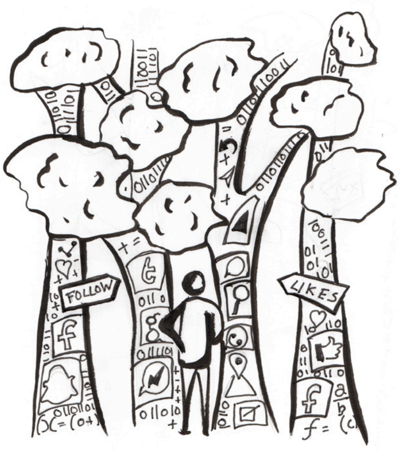

DIGITAL FOREST

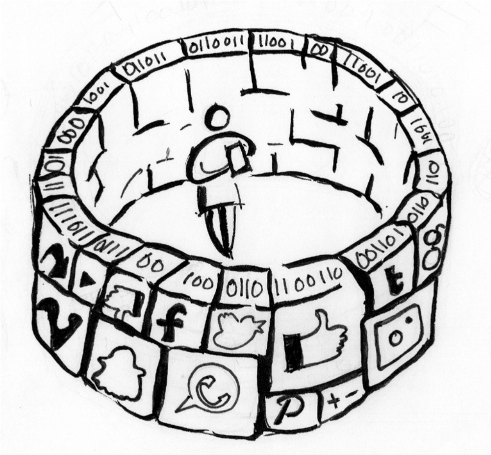

ISOLATION

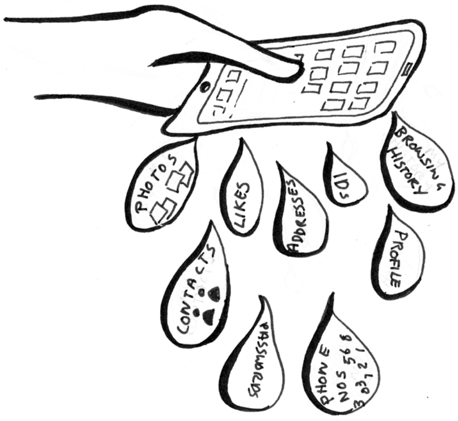

LEAKING DATA

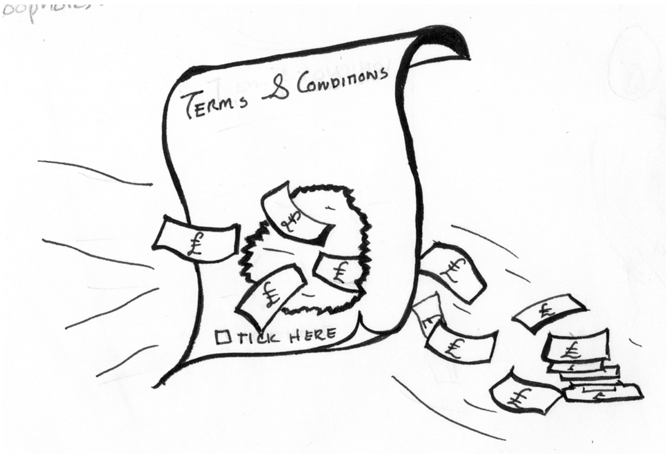

LOOPHOLES

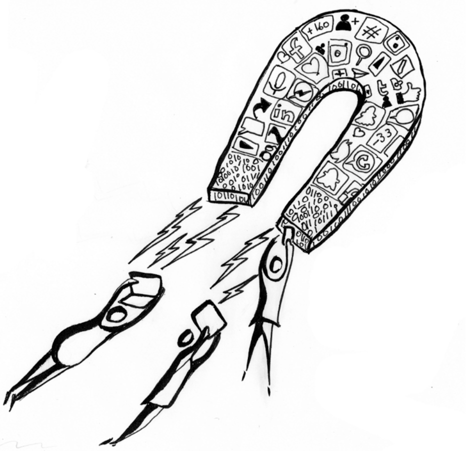

MAGNET

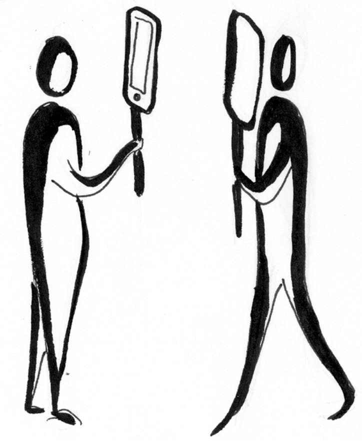

MASK

This work is licensed under a Creative Commons: Attribution Non-Commercial Share-Alike 4.0 International Licence.

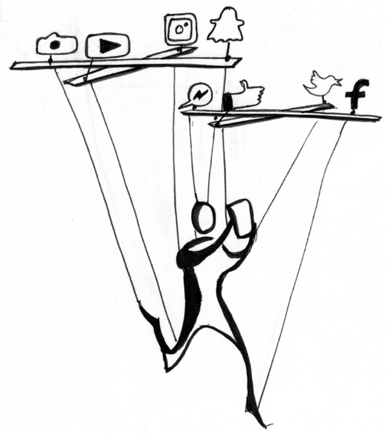

PUPPET

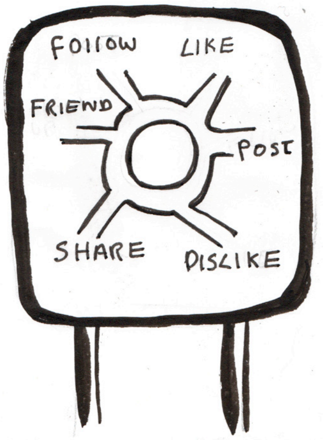

SIGNPOST

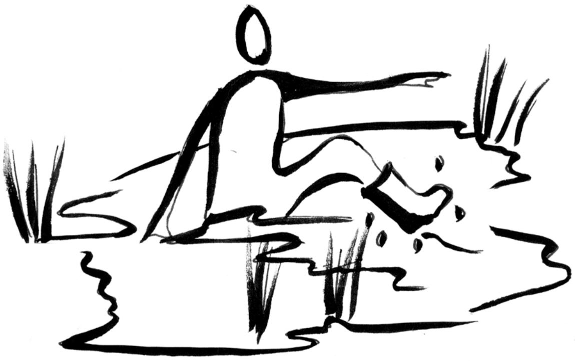

OUT OF DEPTH

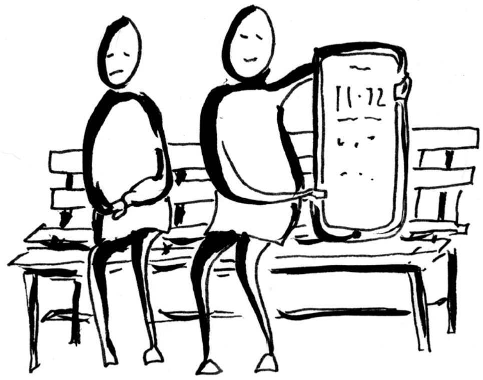

DIGITAL COMPANION

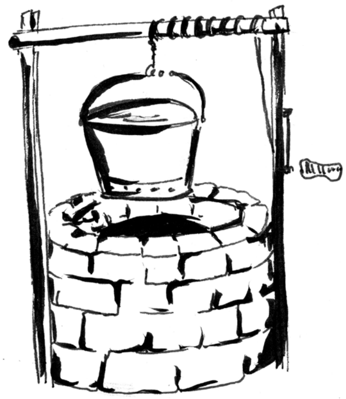

WELL OF DATA

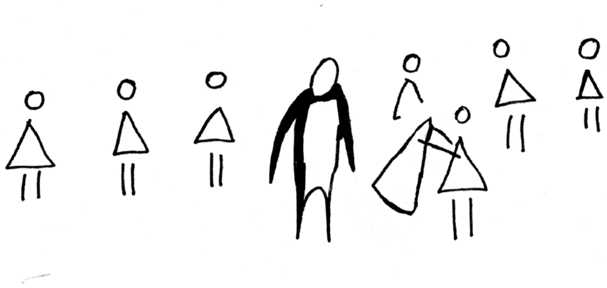

FORCED TO FIT A PROFILE

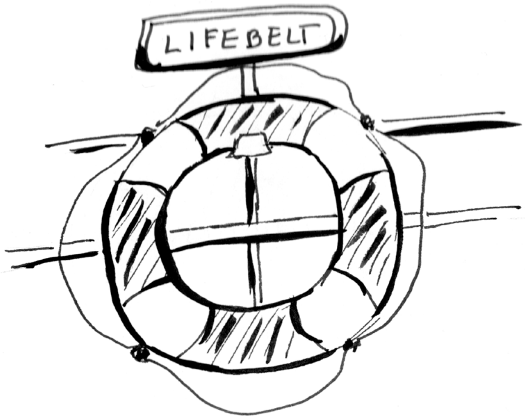

DIGITAL LIFEBELT

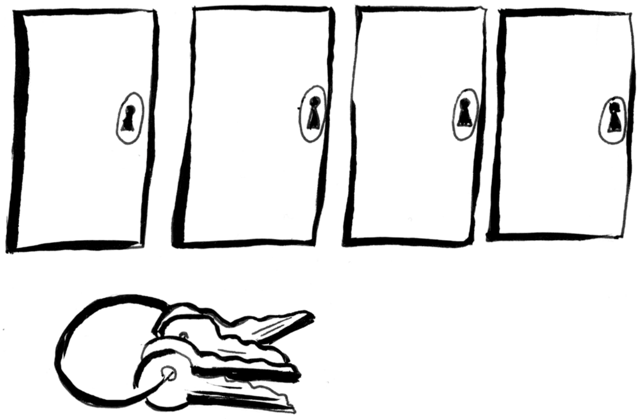

SECURITY

UNBIAS

This work is licensed under a Creative Commons: Attribution Non-Commercial Share-Alike 4.0 International Licence.

Concept & Design
proboscis.org.uk

proboscis
